# Gamification increases tuberculosis awareness in schools from Catalonia: a multi-centre quasi-experimental pre-post intervention study

**DOI:** 10.3389/fpubh.2026.1832611

**Published:** 2026-05-05

**Authors:** Mariona Cortacans, Diego Aznar, Pablo Soldevilla, Maria Vidal, Kaori L. Fonseca, Cristina Vilaplana

**Affiliations:** 1 Experimental Tuberculosis Unit (UTE), Institut de Recerca Germans Trias i Pujol, Badalona, Spain; 2 Department of Genetics and Microbiology, Universitat Autonoma de Barcelona, Barcelona, Spain; 3 Centro de Investigacion Biomedica en Red Enfermedades Respiratorias, Madrid, Spain; 4 Microbiology Department, Northern Metropolitan Clinical Laboratory, Hospital Universitari Germans Trias i Pujol, Badalona, Spain

**Keywords:** board game, educational intervention, gamification, public health, tuberculosis awareness

## Abstract

**Background:**

Tuberculosis (TB) remains one of the world’s deadliest infectious diseases and continues to be hindered by poor public awareness, stigma, and misinformation, all of which can contribute to delayed diagnosis, ongoing transmission, and poor treatment adherence. In low-incidence settings, limited public attention to TB may further reduce basic knowledge and represent an additional barrier to control efforts. We evaluated the feasibility and educational potential of ‘*Tuberculosis Alert*’, the first board game specifically designed around TB, as a gamification tool to support TB awareness among adolescents in a low-incidence country.

**Methods:**

We conducted a quasi-experimental, multicentre pre-intervention/post-intervention study with follow-up among 370 students aged 14–17 years from diverse settings across Catalonia, Spain. The educational intervention was based on ‘*Tuberculosis Alert*’, and its association with TB-related knowledge was assessed using questionnaires administered before, after, and 3–5 months after the intervention. Ordinal logistic regression was used to evaluate changes in score categories after the intervention.

**Results:**

Ordinal logistic regression showed a shift in the score distribution towards higher performance categories after the intervention, with participants having significantly higher odds of achieving a higher score category after playing ‘*Tuberculosis Alert*’ (OR = 19.76, 95% CI 13.88–28.57, *p* < 0.001). Follow-up scores remained above baseline, although not all follow-up comparisons reached statistical significance. Exploratory subgroup analyses did not reveal clear or robust relevant differences according to age, gender, or school area socioeconomic context.

**Conclusion:**

‘*Tuberculosis Alert*’ may be a feasible and useful educational tool to support TB awareness and knowledge acquisition among adolescents in low-incidence settings. These findings support the potential value of gamification-based strategies for TB education, although further controlled studies are needed to confirm their effectiveness and long-term impact.

## 1 Introduction

Tuberculosis (TB) is one of the world’s deadliest infectious diseases, causing over 10 million new cases and 1.25 million deaths each year (1). Despite being treatable, TB disproportionately affects vulnerable and low-income populations, especially in Southeast Asia and Central-Southern Africa. Beyond its health impact, the disease carries heavy social and economic consequences, often worsened by stigma and misinformation.

One of the main obstacles in the fight against TB is the widespread lack of basic knowledge about the disease, including transmission, key symptoms, and preventive measures. Low TB knowledge is linked with disease spread delayed diagnosis, and poor treatment adherence. Importantly, multiple studies report this lack of basic knowledge in high-incidence areas (2–4), further aggravating their situation. In contrast, limited public attention to TB in low-incidence areas often results in insufficient knowledge, posing a significant barrier to TB control efforts in these areas (4). Although Spain is considered a low-incidence country for TB, with fewer than 10 cases per 100,000 population, geographical differences exist. In Catalonia, where case ascertainment is considered reliable, the reported incidence is 15.2 cases per 100,000 population, and the distribution of TB cases shows considerable geographical variability, ranging from 1.4 to 22.4% (5).

Stigma is also a great challenge. TB-related stigma isolates patients from social support and often discourages them from seeking proper medical care and follow-up, worsening TB epidemiology (6). Several factors are associated with stigma in TB, including age, gender, and the level of family and social support. However, the most significant factor is the level of knowledge about the disease. Multiple studies have shown that lower awareness and understanding of TB is linked to higher self-stigma and externally perceived stigma (7–9).

The development of new educational tools offers an opportunity to facilitate public access to basic knowledge about TB in a more approachable and inclusive way. In this regard, gamification—the application of game elements and mechanics to educational or awareness-raising contexts-emerges as an effective approach to achieve this goal. This methodology enables participants to take an active role in the learning process, thereby reinforcing knowledge acquisition. Moreover, it has proven effective-especially among younger audiences (children and adolescents)—in disseminating knowledge across various domains, including infectious diseases (10, 11).

In this context, we developed *‘Tuberculosis Alert’*, a cooperative board game that simulates a TB outbreak and represents the first board game focused on TB. We hypothesise that this gamification tool can help improve general knowledge about TB among the general public, particularly adolescents. Accordingly, this study aims to investigate whether the ‘*Tuberculosis Alert’* is an effective gamification tool for enhancing TB awareness among adolescents across several regions in Catalonia.

## 2 Methods

### 2.1 Study design and ethics

‘*Tuberculosis Alert’* is a cooperative educational board game developed by members of the Experimental Tuberculosis Unit (UTE) from the Germans Trias i Pujol Research Institute (IGTP), which holds all intellectual property rights. The game is centred around a game board and includes role, action, gamemaster and wild cards, and markers indicating disease progression in the lungs, outbreak expansion, and elapsed time. All text components are available in Catalan, Spanish, English, and French. The study was conducted in Catalonia, Spain, between September 2024 and May 2025. Schools were recruited through a public call for participation disseminated via social media. Based on responses, we co-designed the intervention with selected schools across several municipalities. We conducted a quasi-experimental, multicentre pre-intervention/post-intervention study with follow-up to evaluate the impact of *‘Tuberculosis Alert’* on adolescents’ knowledge of TB.

Ethical approval was not required for this study in accordance with applicable Spanish and institutional regulations. The study consisted exclusively of an educational intervention—a board game session integrated into regular school hours—combined with the administration of anonymous questionnaires. No medical procedures, biological samples, clinical interventions, or personal identifiers were collected at any point. Under Spanish Law 14/2007 on Biomedical Research, mandatory ethics committee review applies to biomedical research involving clinical human interventions or biological samples of human origin; this study does not fall within that scope. The study was conducted in full accordance with the principles of the Declaration of Helsinki, specifically its provisions on minimal-risk research, and with Organic Law 3/2018 (LOPDGDD) on personal data protection, under which participants aged 14 and above may provide consent for the processing of their own anonymised data in observational, non-invasive research contexts. Institutional responsibility for the activity rested with the participating schools, whose authorities provided verbal consent for implementation. All participants provided oral consent prior to participation, and all data were collected anonymously and analysed only in aggregate.

### 2.2 Participants

A total of 370 students aged 14–17 years from nine schools across seven municipalities in Catalonia participated. An open call was disseminated to secondary schools across Catalonia through social media. The intervention was offered over one academic year (September to July), and all schools that expressed interest were included and scheduled for participation. The resulting sample can therefore be considered a self-selected convenience sample based on schools’ willingness to participate, with one additional school included through collaboration with the regional public health department as part of a TB contact tracing study. The activity was integrated into the schools’ regular schedules, with ethical responsibility for the implementation resting with the schools. Participation was voluntary, with verbal institutional consent obtained from school authorities and individual oral consent obtained from participants at the start of each session. No direct personal identifiers (such as names or contact information) were collected, and all data were anonymised. Self-reported demographic variables (age, gender, and place of residence) were analysed in aggregate to ensure confidentiality. Inclusion criteria were enrolment in a participating school and attendance on the intervention day. No specific exclusion criteria were applied.

### 2.3 Questionnaire development and scoring

A study-specific questionnaire was developed by the research team to assess knowledge of the main TB concepts addressed by the game. The questionnaire was developed specifically for this study by the research team. Before implementation, it was informally piloted with individuals familiar and unfamiliar with TB to assess item clarity, comprehensibility, and appropriateness for the target population. Minor wording adjustments were made before use in the study. The instrument consisted of 10 multiple-choice items. This questionnaire included both general questions (e.g., what TB is, how it is transmitted, its symptoms, who can be infected, and how it can be prevented) and more specific questions (e.g., current treatment, treatment duration, the implications of multidrug-resistant TB (MDR-TB), and the concept of latency). The questionnaire was anonymous and voluntary. Self-reported information on age, gender, and place of residence was also collected (Annex 1). Each item had one correct answer and was scored as 1 for a correct response and 0 for an incorrect or missing response. A total knowledge score was calculated as the sum of correct answers, ranging from 0 to 10. The same knowledge items were administered before and immediately after the intervention, and again at follow-up when possible. Internal consistency of the pre-intervention knowledge questionnaire was assessed using the Kuder–Richardson coefficient (KR-20).

### 2.4 Procedures

**Figure 1:**
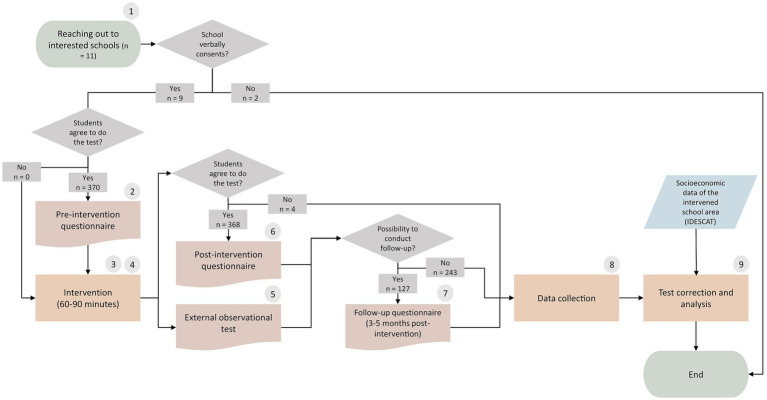
Flowchart of the study procedures. A total of 370 students participated in the intervention. Pre- and post-intervention questionnaires were completed by 370 and 368 students, respectively. The 3–5-month follow-up questionnaire was completed by 127 students (34.4%). Follow-up was only conducted in only four of the nine participating schools due to logistical constraints related to the school calendar. IDESCAT, Statistical Institute of Catalonia.

**Figure 2:**
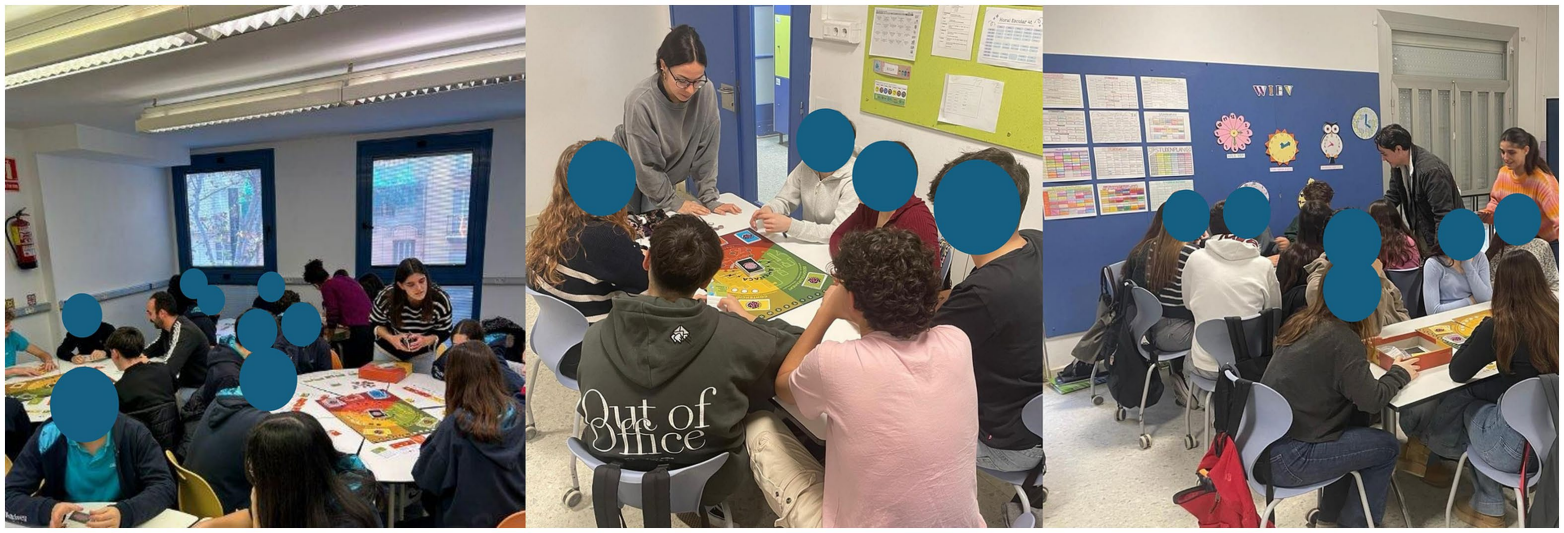
Photographs of the *‘Tuberculosis Alert’* intervention in participating schools. Images depict students engaging in gameplay during the intervention sessions across different schools in Catalonia. The photographs illustrate the implementation of the board game in classroom settings, including group organisation, facilitator guidance, and the use of the game board and components during play. The board game was used to simulate a TB outbreak scenario and foster interactive learning. Activities were carried out in groups of 4–6 students and supervised by members of the research team (visible in the photograph), typically during regular school hours. All schools pictured provided verbal consent for the use of anonymised photographs for scientific dissemination purposes.

Each visit was coordinated with school staff and conducted during regular school hours following a structured sequence (Figure 1). Prior to intervention day (1), verbal consent was obtained from participating educational centres, and logistical details were agreed upon with the researchers. (2) Immediately before the intervention, students completed a voluntary, anonymous pre-intervention questionnaire to assess baseline knowledge. Self-reported information on age, gender, and place of residence was also collected (Annex 1). (3) Members of the research team, who were also the developers of the board game, introduced the activity and explained its objectives and rules following the same standardised procedure. (4) Students played *‘Tuberculosis Alert’* in groups of four to six, each guided by one of the facilitators (Figure 2). During gameplay, participants interacted with the game master, which structured the progression of the simulated TB outbreak and guided group-based decision-making throughout the session. Sessions lasted approximately 60–90 min, including a brief discussion and solving participant questions. (5) An external observer (typically a teacher) assessed session organisation, difficulty, student engagement, social skills promotion, and educational value using a five-point Likert scale (Annex 2). (6) Immediately after gameplay, students completed a post-intervention questionnaire containing the same knowledge items as the pre-intervention questionnaire, as well as a satisfaction question, also rated on a five-point Likert scale. (7) To assess long-term knowledge retention, a follow-up questionnaire was administered 3–5 months later by schoolteachers. All questionnaires were completed individually under supervision. (8) Paper questionnaires were collected, and responses were entered into an Excel database. (9) Finally, to evaluate the effectiveness of the game across different social environments, results were stratified according to the socioeconomic index (SEI) of each school’s location, the type of educational institution (public, semi-private, or private), and the age and gender of participants. The SEI, provided by the Statistical Institute of Catalonia (IDESCAT), is a composite indicator of employment, education, immigration rates, and income (12). A value of 100 represents the Catalan average. For analytical purposes, schools were categorised as falling above or below this study’s SEI median.

The primary outcome of the study was the change in TB-related knowledge among participating students, assessed through pre- and post-intervention questionnaires and a follow-up questionnaire administered 3–5 months later. This outcome was measured in all students who completed both the pre- and post-questionnaires. Secondary outcomes included students’ self-reported satisfaction with the activity and the external observer’s assessment of session quality.

### 2.5 Statistical analysis

Data analysis was conducted with R (version 4.5.1) and R studio (version 2025.05.1). The following R packages were used for data management, analysis, and visualisation: readxl (v. 1.4.5) (13), sf (1.0–21) (14), gtsummary (v. 2.2.0) (15), ggplot2 (v. 3.5.2) (16), ggbeeswarm (v. 0.7.2) (17), ggpubr (v. 0.6.0) (18), tidyr (v. 1.3.1) (19), dplyr (v. 1.1.4) (20), rstatix (0.7.2) (21), MASS (7.3–65) (22), and brant (0.3–0) (23). The primary outcome was the change in TB-related knowledge following the intervention. Data normality was assessed prior to analysis using the Shapiro test and Q-Q plots. Paired two-sided Wilcoxon tests were used to compare pre- and post-intervention scores as median scores by schools. Paired Wilcoxon effect sizes were computed, and since the study was designed to test a single primary comparison, no adjustment for multiple comparisons was applied to the primary analysis. An ordinal logistic regression model (24) (proportional odds) was used to estimate odds ratios (ORs) and 95% confidence intervals (CIs) for achieving a higher score category after the intervention based on the grading scale used in the participants’ educational system (≤4 = Fail, 5–6 = Pass, 7–8 = Good, 9–10 = Excellent). The primary predictor was the intervention (pre- and post-intervention). Initially, age and gender were considered as potential covariates. Likelihood ratio tests were used to evaluate whether inclusion of these covariates improved model fit. Model assumptions and stability were assessed using the Brant test and Akaike Information Criterion (AIC). Model-based predicted probabilities were computed to facilitate interpretation and visualization of effects. Exploratory secondary analysis included assessing the effect of each school’s SEI, students’ satisfaction with the activity, and external observers’ ratings of the session. To assess the effect of schools’ SEI, unpaired two-sided Wilcoxon tests were used to compare questionnaire scores across school areas with SEIs above or equal to the sample median and SEIs below the sample median. Associations between continuous variables (median age, percentage of female students, and schools’ SEI) and pre- and post-intervention questionnaire scores were analysed using Spearman’s correlation and did not account for missing values. Analyses of secondary outcomes were considered exploratory, and *p*-values should be interpreted with caution.

## 3 Results

### 3.1 Demographic characteristics of the participants

The intervention was implemented across nine schools in seven municipalities in the provinces of Barcelona and Girona, Catalonia, Spain (Table 1; Supplementary Figure 1). A total of 370 students aged 14–17 years participated in the study. Gender was self-reported by 359 of 370 participants. Among those who responded, 189 (51.1%) identified as female and 170 (45.9%) as male. Eleven students (3%) did not report their gender. Only one game session was conducted among upper-secondary (baccalaureate) students (*n* = 18); the remainder involved fourth-year students of compulsory secondary education (*n* = 352).

**Table 1:** Demographic data of study participants (*n* = 370).

Characteristic	Pre-intervention *N* = 370^1^	Post-intervention *N* = 368^1^	3–5 months post-intervention *N* = 127*^1^*
Age			
14	20 (5.4%)	22 (6%)	0 (0%)
15	167 (45.1%)	167 (45.4%)	46 (36.2%)
16	145 (39.2%)	141 (38.3%)	54 (42.5%)
17	37 (10%)	35 (9.5%)	27 (21.3%)
No answer	1 (0.3%)	3 (0.8%)	0 (0%)
Gender			
Female	189 (51.1%)	186 (50.5%)	68 (53.5%)
Male	170 (45.9%)	167 (45.4%)	59 (46.5%)
No answer	11 (3%)	15 (4.1%)	0 (0%)
Students per school grade			
4th ESO	352 (95.1%)	350 (95.1%)	127 (100%)
Baccalaureate	18 (4.9%)	18 (4.9%)	0 (0%)
Students per school type			
Public	96 (25.9%)	94 (25.5%)	54 (42.5%)
Semi-private	137 (37%)	135 (36.7%)	73 (57.5%)
Private	137 (37%)	139 (37.8%)	0 (0%)
Students per school municipality			
Badalona	57 (15.4%)	56 (15.2%)	29 (22.8%)
La Jonquera	19 (5.1%)	19 (5.2%)	0 (0%)
Mataró	53 (14.3%)	53 (14.4%)	20 (15.7%)
Montgat	137 (37%)	139 (37.8%)	0 (0%)
Sant Adrià de Besòs	24 (6.5%)	22 (6%)	0 (0%)
Sant Feliu de Codines	27 (7.3%)	26 (7.1%)	24 (18.9%)
Vilassar de Dalt	53 (14.3%)	53 (14.4%)	54 (42.5%)
Students per School Area Socioeconomic Index (SEI)			
Below or equal to median	128 (34.6%)	124 (33.7%)	24 (18.9%)
Above median	242 (65.4%)	244 (66.3%)	103 (81.1%)

^1^*n* (%).

β Gender was self-reported by students aged 14–17 years, with the majority (95.1%) being in the fourth year of compulsory secondary education (ESO). Participants were drawn from nine schools of varying types: three public, five semi-private, and one private. Schools were geographically distributed across seven municipalities in Catalonia, including both urban (e.g., Barcelona Metropolitan Area) and semi-rural regions (e.g., La Jonquera, Alt Empordà). Socioeconomic Index (SEI) values for each school were obtained from the Statistical Institute of Catalonia (IDESCAT) and categorised based on the median SEI value of our dataset (111.4). Five schools were classified as having an SEI at or below the median, and four schools were above the median. Follow-up data (3–5 months post-intervention) were only available for students from four of the nine schools.

Regarding school characteristics, three of the nine schools were public, five were semi-private, and one was private. Four schools were located within the Barcelona Metropolitan Area (Badalona, Sant Adrià de Besòs, and Montgat), whilst the remaining five were in more peripheral municipalities, including Sant Feliu de Codines (Vallès Oriental), Vilassar de Dalt and Mataró (Maresme), and La Jonquera (Alt Empordà, Girona), which borders France.

Based on the SEI of the nine schools’ locations, five schools fell at or below the sample median (111.4), whilst four were above it. Of the total schools, four provided complete data across all three time points (pre-intervention, post-intervention, and 3–5-month follow-up), accounting for 127 students. The remaining five schools lacked follow-up data because the planned 3- to 5-month assessment fell outside the same academic year.

### 3.2 Higher post-intervention scores were observed for both basic and specific TB knowledge, with this pattern persisting at follow-up

**Figure 3:**
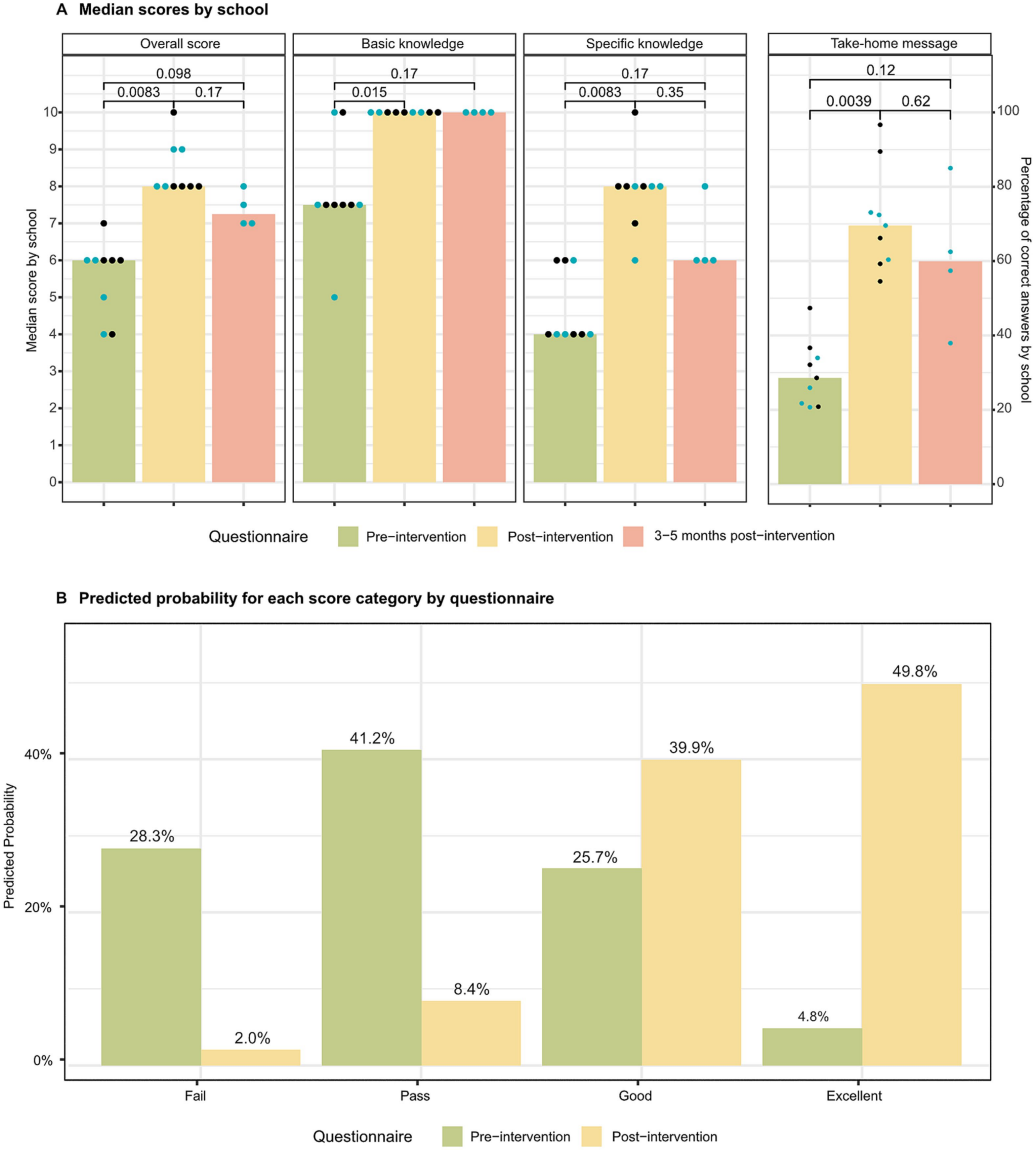
The board game increased TB awareness, with knowledge maintained over time. **(A)** Median scores by school. Bar charts show median score by school for overall, basic and specific TB knowledge, at pre-intervention (green), post-intervention (yellow), and 3–5 months post-intervention (salmon/pink). The panel on the right contains bar chart showing the percentage of correct answers to the take-home message question by school, at pre-intervention (green), post-intervention (yellow), and 3–5 months post-intervention (salmon/pink). For the follow-up questionnaire comparisons, the four schools that have data for all three questionnaires were considered (blue dots). Wilcoxon two-sided paired test. Significance levels: *p < 0.05; **p < 0.01; ns, non-significant. *N* = 9 for pre-intervention questionnaire scores vs. post-intervention scores. *N* = 4 for comparisons with 3–5 months post-intervention. **(B)** Predicted probability for each score category by questionnaire. Predicted probability for score categories (0–4 = Fail, 5–6 = Pass, 8–7 = Good, 9–10 = Excellent), computed with an ordinal logistic regression model of proportional odds for both the pre-intervention (green), and the post-intervention (yellow) questionnaires. *N* = 370 for pre-intervention questionnaire and *n* = 368 for post-intervention questionnaire.

In each intervention, participants’ TB-related knowledge was assessed using questionnaires administered immediately before and after the activity. These covered both basic and specific concepts. This questionnaire was assessed for internal consistency using the KR-20 test at baseline, yielding a value of 0.42. Post-intervention results from 368 student questionnaires showed statistically significant differences across all sections compared to baseline: overall scores increased by two points, basic knowledge by 2.5, specific knowledge by four, and correct answers to the take-home message question rose to 70% (Figure 3A). A follow-up questionnaire administered three to 5 months later in a subset of four schools showed that scores remained higher than baseline, although not all differences reached statistical significance; however, as shown in Table 2, these pairwise comparisons were generally associated with large effect sizes across overall, basic, and specific knowledge domains. Due to end-of-year constraints, this follow-up could not be conducted in five of the nine schools. No significant differences were observed between the immediate post-intervention and the 3- to 5-month scores, with basic knowledge remaining stable.

**Table 2:** Effect sizes for score comparisons.

Comparison	Comparison group 1	Comparison group 2	Effect size	Magnitude
Overall score				
	Pre-intervention (*n* = 9)	Post-intervention (*n* = 9)	0.900	Large
	Pre-intervention (*n* = 4)	3–5 months post intervention (*n* = 4)	0.921	Large
	Post-intervention (*n* = 4)	3–5 months post intervention (*n* = 4)	0.843	Large
Basic knowledge				
	Pre-intervention (*n* = 9)	Post-intervention (*n* = 9)	0.864	Large
	Pre-intervention (*n* = 4)	3–5 months post intervention (*n* = 4)	0.707	Large
	Post-intervention (*n* = 4)	3–5 months post intervention (*n* = 4)	N/A	N/A
Specific knowledge				
	Pre-intervention (*n* = 9)	Post-intervention (*n* = 9)	0.900	Large
	Pre-intervention (*n* = 4)	3–5 months post intervention (*n* = 4)	0.843	Large
	Post-intervention (*n* = 4)	3–5 months post intervention (*n* = 4)	0.707	Large
Take-home message				
	Pre-intervention (*n* = 9)	Post-intervention (*n* = 9)	0.889	Large
	Pre-intervention (*n* = 4)	3–5 months post intervention (*n* = 4)	0.913	Large
	Post-intervention (*n* = 4)	3–5 months post intervention (*n* = 4)	0.365	Moderate

Wilcoxon paired effect sizes were calculated with the wilcox_effsize() function of the rstatix package (0.7.2). Effect size magnitudes are classified as: 0–< 0.3 = small effect, 0.3 – <0.5 = moderate effect, > = 0.5 = large effect.

To further assess the increase in TB-associated knowledge, participant scores were categorised into the standard grading scale of the Spanish educational system (0–4 = Fail, 5–6 = Pass, 8–7 = Good, 9–10 = Excellent). An ordinal logistic regression model of proportional odds indicated that the intervention was a strong predictor of participants’ score category, with participants having significanly higher odds of being in a higher score category compared to before the intervention (OR = 19.76, 95% CI [13.88–28.57], *p* < 0.001). Likelihood ratio testing suggested a weak association between age and performance. However, this effect was not robust and did not improve model fit meaningfully, whereas gender was not associated with performance at all in our data. Moreover, predicted probabilities of being in each score category before and after playing *‘Tuberculosis Alert’* (Figure 3B) showed a shift in outcome distribution. In the pre-intervention assessment, the highest predicted probabilities were concentrated in the lower categories, particularly Pass (41.2%) and Fail (28.3%), whereas Excellent was uncommon (4.8%). In contrast, in the post-intervention assessment, the distribution shifted towards the higher categories, with Excellent becoming the most likely outcome (49.8%) and Good also increasing (39.9%), whilst the probabilities of Pass and Fail decreased to 8.4 and 2.0%, respectively. Overall, these results indicate a marked shift from lower to higher score categories after the intervention.

### 3.3 Exploratory analyses according to demographic and contextual variables showed no differences in TB knowledge scores, and satisfaction ratings were high

Exploratory *post hoc* analyses were performed to examine whether questionnaire scores varied according to age, gender, or the area-level SEI of each school’s location. No significant differences in pre- or post-intervention scores were observed according to SEI, and correlation analyses showed no significant association between SEI and questionnaire scores (Supplementary Figure 2). Age showed a non-significant trend with baseline scores (*p* = 0.17), but this was not observed in the post-intervention assessment (*p* = 0.92), suggesting that age was not linked to differential learning outcomes following the intervention. No significant differences were observed according to gender in either the pre- or post-intervention questionnaires (*p* = 0.43 and *p* = 0.089, respectively). As these analyses were exploratory and were not part of the primary study objective, they should be interpreted with caution, and no firm conclusions can be drawn from them. Acceptability findings were favourable, with high satisfaction ratings from both students and external observers. Among participants who completed the post-intervention questionnaire (*n* = 368), 303 (82.3%) answered the satisfaction question. Of those 303, 199 (65.7%) rated the game with the highest score (five out of five), 91 (30%) rated it a four, 11 (3.6%) rated it a three, and 2 (0.66%) rated it a two. The remaining 65 (21.5%) participants omitted the satisfaction question. Among external observers (*n* = 10), six (60%) gave a rating of five, whilst the remaining scores ranged between four and 4.8. Overall, these results suggest that the intervention was positively received in both educational and observational contexts.

## 4 Discussion

This study evaluated the feasibility and educational potential of *‘Tuberculosis Alert’*, a cooperative educational board game, as a gamification strategy to improve TB awareness among adolescents in a low-incidence country. The intervention was associated with increased TB-related knowledge scores, including a shift in score distribution towards higher performance categories, as well as positive evaluations from students and teachers. These findings support the potential of gamified, school-based tools to promote TB education in adolescent populations.

To our knowledge, *‘Tuberculosis Alert’* is the first board game specifically designed to address TB knowledge in the general population. Previous gamification tools on infectious diseases, such as *‘Outbreak!’, ‘SARS Wars’*, and ‘*Lockdown!’* have explored engagement and educational potential but did not assess knowledge acquisition directly (25–27). Other tools do include pre- and post-intervention questionnaires but are intended for healthcare professionals (e.g., ‘*AntimicroGAME’*, ‘*AMS Game’*) (28). In addition, many existing games are not cooperative or focus on fictional or non-specific disease scenarios (e.g., ‘*Zombiepox’*, ‘*Pandemic*’, ‘*Virus*’) (25). In this context, ‘*Tuberculosis Alert’* addresses a clear gap by combining disease-specific content, a cooperative format, and evaluation of educational outcomes in a general adolescent population.

Although TB-specific educational interventions in adolescents remain limited, some school-based studies have reported higher TB-related knowledge after educational activities. For example, a pre−/post-intervention study in secondary-school students in Gambia (29) reported improved TB knowledge and awareness following an educational intervention, whilst a quasi-experimental study in Thai high-school students (30) found higher TB knowledge together with improvements in attitudes, self-efficacy, and stigma-related outcomes after a communication-based programme. More broadly, game-based and gamified tools have also shown educational value in other infectious-disease contexts, including a board game on enterovirus for schoolchildren (31), a gamification-based leptospirosis (32) intervention in university students, and a serious game designed to increase adolescents’ awareness of health and security risks in an Italian sample (33). Although these interventions differ from our cooperative board game and are not TB-specific, they support the broader relevance of interactive educational approaches in young populations.

The cooperative nature of the game may have contributed to its educational value. Cooperative gamification has been associated with collaborative learning, peer communication, social interaction, and decision-making, as described by Gustavo de Almeida et al. (2021). Consistent with these findings, general-audience games such as *‘MIApp’*, *‘*Stop the Spread*’*, and a cutaneous leishmaniasis prevention game have also shown measurable improvements in topic-specific knowledge (25, 34). However, we were unable to identify recent studies quantifying TB awareness or knowledge in the general population, particularly in low-incidence settings such as Spain. This remains an important gap, although recent work from northern Italy has highlighted the continued relevance of measuring TB-related knowledge in low-incidence European contexts through the development and validation of a TB questionnaire in migrant populations (35). An important strength of this study is the inclusion of follow-up data, which suggests that higher knowledge scores may be maintained over time. This is particularly important given that long-term awareness is essential for encouraging timely health-seeking behaviours and adherence to treatment. Although we did not assess behaviour, similar gamification-based interventions have shown promise in improving health-related intentions and self-efficacy (36). Future studies should evaluate whether the higher scores observed after participation in *‘Tuberculosis Alert’* translate into measurable behavioural changes over longer timeframes.

In addition to the main analyses, we explored whether questionnaire scores varied according to age, gender, and the schools’ SEI. Previous literature has described associations between socioeconomic disadvantage and lower health literacy (37, 38), however, our exploratory analyses did not identify consistent differences in our dataset, nor did they allow firm conclusions on this issue, as the study was not specifically designed to investigate the mechanisms underlying socioeconomic differences in learning outcomes, and alternative explanations may account for the observed findings. Satisfaction findings, however, consistently supported the acceptability and feasibility of the intervention, with highly positive evaluations from students, teachers, and observers.

The main limitation of this study is the absence of a control group (e.g., schools receiving standard TB lectures or no intervention), which prevents causal attribution of the observed score differences to the board game. Although post-intervention and follow-up scores were higher than baseline, alternative explanations cannot be excluded, including a testing effect due to repeated questionnaire administration, social desirability bias, and short-term engagement or novelty effects related to the intervention context. Moreover, follow-up data were available for only a subset of schools, which may limit representativeness and introduce potential attrition bias. Therefore, the findings should be interpreted as showing an association between participation in the intervention and higher questionnaire performance, including a shift towards higher score categories, rather than definitive evidence of effectiveness.

In addition, the questionnaire was developed specifically for this study and was not previously validated. However, it was piloted before implementation to ensure clarity. When assessing reliability, the questionnaire showed modest internal consistency at baseline (KR-20 = 0.42), likely reflecting its brief nature and the inclusion of items covering multiple domains of TB-related knowledge rather than a single construct. This is not unexpected for a brief knowledge test covering multiple content domains rather than a single underlying construct. Given that TB incidence in Spain averages 5.9 cases per 100,000 inhabitants (1) but is notably higher in vulnerable areas (39), future research should replicate this intervention across diverse settings and consider longer or remote follow-up strategies.

Overall, our findings suggest that *‘Tuberculosis Alert’* may be a useful educational tool for TB awareness among adolescents in a low-incidence setting. The intervention appears feasible, well accepted, and may support knowledge acquisition in adolescents. Beyond school settings, ‘*Tuberculosis Alert’* may also have value in community outreach, awareness campaigns, or screening-related educational activities across different TB incidence settings. Its use may also be explored within TB drug and vaccine projects to support engagement and communication with affected individuals, families, and communities. Future research should assess these broader applications and examine how gamified education can be integrated into public health and community-based TB strategies.

## Data Availability

The raw data supporting the conclusions of this article will be made available by the authors, without undue reservation.
