# Supplementary material for: Gamification increases tuberculosis awareness in schools from Catalonia: a multi-centre quasi-experimental pre-post intervention study
Source: Front Public Health. 2026 May 5;14:1832611. doi: 10.3389/fpubh.2026.1832611 (PMC13183655; doi:10.3389/fpubh.2026.1832611)
Supplement: Supplementary file 1 [file Data_Sheet_1.docx]

**Supplementary Data**


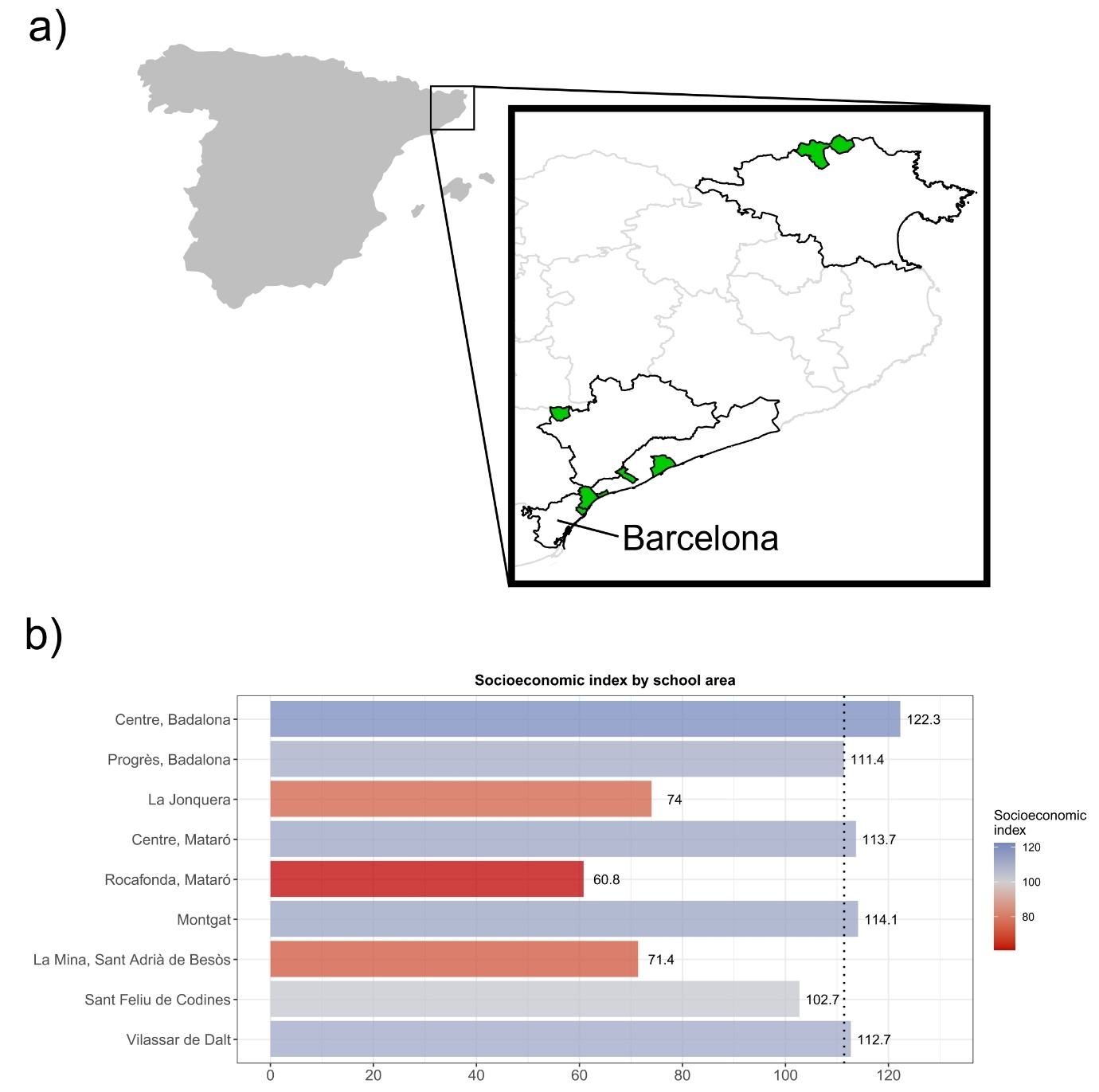


**Supplementary Figure 1. Municipalities and school area socioeconomic index.** Panel a) depicts a map of Spain with the Catalan municipalities (highlighted in green) and their respective shires (black outline) where the game sessions were conducted. Panel b) shows the socioeconomic index (SEI) of the intervened school areas. Bar height represents the SEI value for each school area. The dotted line represents the median (111.4) for our data. Colour scales midpoint is centred to the base SEI of Catalonia (100). The GeoJSON files were downloaded from https://github.com/ArnauInes/geometries_cat_bcn_2024.


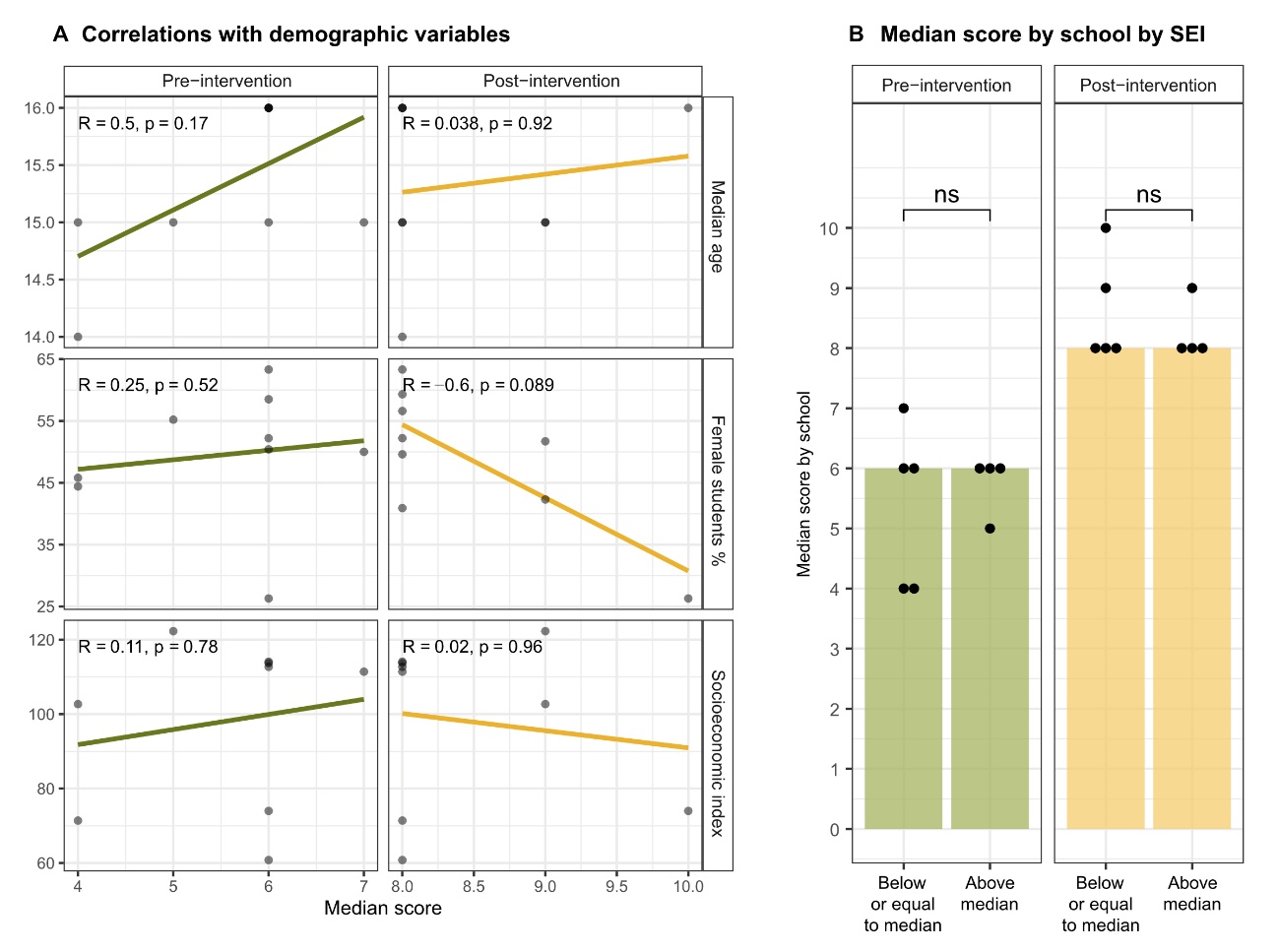


**Supplementary Figure 2. A. Correlations between demographic variables and knowledge scores.** Each point represents one school. Median questionnaire scores (pre- and post-intervention) are plotted against the median age, percentage of female students, and socioeconomic index (SEI) of each school. Spearman’s correlation coefficients (ρ) were calculated at the school level to assess associations between demographic variables and knowledge scores. Significance levels: p < 0.05. **B. Median score by school by socioeconomic index (SEI).** Median questionnaire scores per school, stratified by the SEI of the school’s geographic area. Bar height indicates the median across all schools. Statistical comparisons were made using the two-sided unpaired Wilcoxon test. ns indicates non-significant differences.


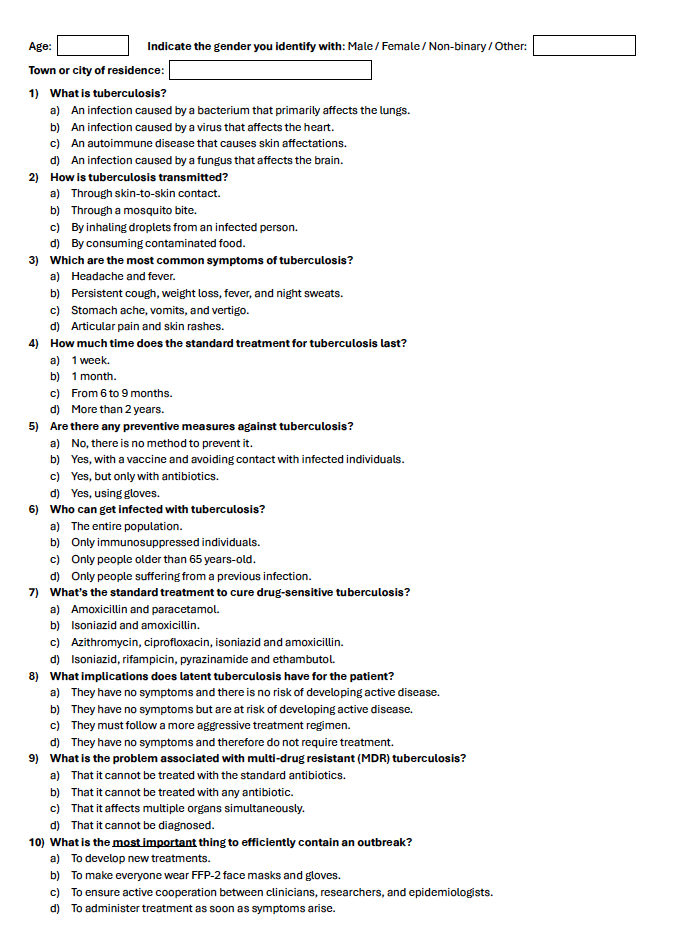


**Annex 1. Pre- and post-intervention questionnaire used to assess TB knowledge.** The questionnaire was administered immediately before and after the intervention to evaluate participants’ knowledge of TB. It included general knowledge items (e.g., transmission, symptoms, prevention) and specific items (e.g., treatment duration, multidrug-resistant TB, latency). The same questions were used in both the pre- and post-intervention assessments. A follow-up identical version was administered three to five months later to evaluate knowledge retention.


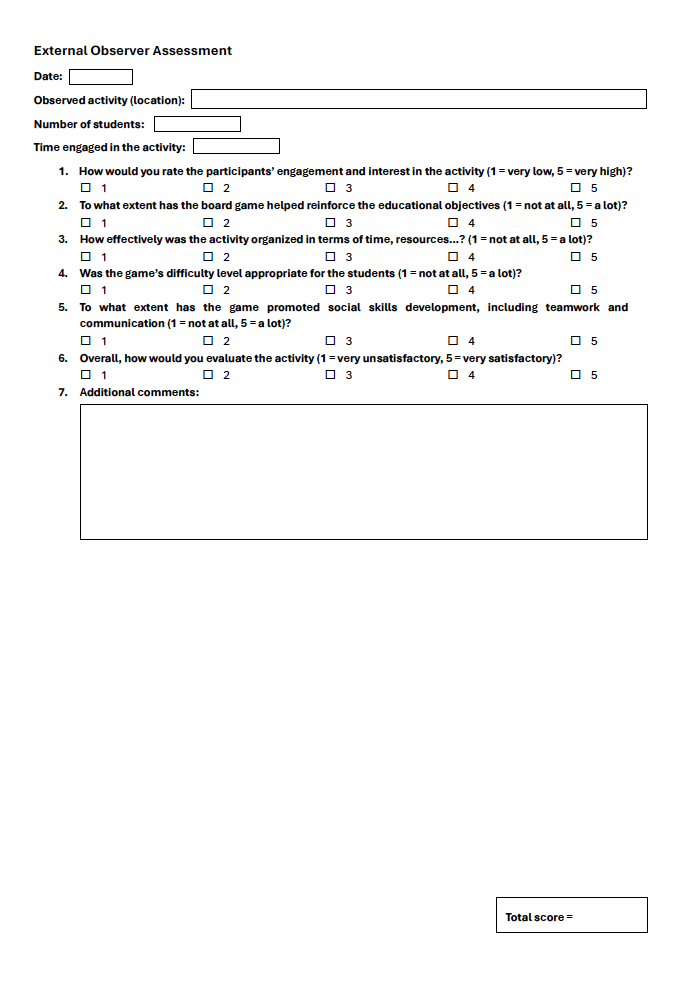


**Annex 2. External observer assessment of the intervention sessions.** The figure shows the evaluation form used by external observers during the intervention. Observers rated the quality of each session using a five-point Likert scale (1 = very poor, 5 = excellent) across multiple dimensions: logistical organisation, difficulty level, student engagement, promotion of social skills, and alignment with educational goals. These evaluations were used to assess the perceived quality and feasibility of the *‘Tuberculosis Alert’* board game intervention in school settings.
